# The Association of Dysfunctional Attitudes and Adult Separation Anxiety in Pregnancy: A Cross-Sectional Study

**DOI:** 10.7759/cureus.42025

**Published:** 2023-07-17

**Authors:** Merve Akkus, Fatih Akkuş

**Affiliations:** 1 Psychiatry, Kütahya Health Science University, Kütahya, TUR; 2 Obstetrics and Gynaecology, Necmettin Erbakan Üniversity Meram Medical School, Konya, TUR

**Keywords:** maternal mental health, woman, cognitive theory, pregnancy, dysfunctional attitudes, adult seperation anxiety

## Abstract

Background

This study aims to examine adult separation anxiety in pregnant women and its relationship with dysfunctional attitudes from the perspective of cognitive theory.

Methods

The sociodemographic data, Adult Separation Anxiety Questionnaire (ASA-27), and Dysfunctional Attitudes Scale-Revised (DAS-R) questionnaire were utilised to examine the correlation between sociodemographic factors and the ASA and dysfunctional attitudes in women attending antenatal obstetric clinics.

Results

The study included 190 pregnant women. Of these, 45.8% (n=87) had significant adult separation anxiety disorder (ASAD) symptoms. There was no significant difference between the ASAD (+) and ASAD (-) groups in terms of sociodemographic characteristics (p>0.05). However, a significant association was found between ASAD and the gestational period (p=0.02). DAS-R scores were higher in patients with ASAD symptoms (p<0.001). Positive correlations were observed between DAS-R and perfectionism or achievement and between DAS-R and the need for approval or dependency (p=0.001). DAS-R and gestational week showed potential as predictors of ASAD positivity (Area under the ROC curve (AUC)=0.725 vs. 0.632, respectively).

Conclusion

Pregnancy can be a sensitive period for the development of separation anxiety in adults. The development and treatment of separation anxiety in adults may benefit from cognitive-behavioural interventions that address dysfunctional attitudes during pregnancy.

## Introduction

Pregnancy is a complex period in which biological, physiological, psychological, and social changes are observed. Although pregnancy is typically considered a period of emotional well-being, recent studies show that 20% of women experience mood changes or anxiety disorders during this period [1]. The main psychiatric disorders during pregnancy are anxiety disorders and depressive disorders [2, 3].

In the Diagnostic and Statistical Manual of Mental Disorders (DSM)-IV and earlier, separation anxiety disorder was evaluated under childhood anxiety disorders [4]. With the DSM-5, the age criterion was removed, and adult separation anxiety was defined as a disease [5]. The lifetime incidence has been reported to be approximately 6% in studies. It is observed more frequently in women than in men [6]. A higher incidence has been reported in the presence of comorbid psychiatric conditions [4].

It has been shown epidemiologically that adult separation anxiety can take two forms. It has been stated that it can start in childhood and continue in adulthood, or it can start for the first time in adulthood. Psychosocial stressors are thought to be effective in the development of separation anxiety disorder in adulthood [4, 7].

In cognitive theory, dysfunctional attitudes are defined as negative beliefs that a person develops against himself, his social environment, and the outside world during communication with others [8]. There are studies reporting the relationship between dysfunctional attitudes and depression and anxiety. Dysfunctional attitudes are known to have a noteworthy function in instigating and intensifying anxiety disorders. Individuals who exhibit elevated levels of dysfunctional attitudes frequently possess an augmented perception of menace and construe situations as being more hazardous or unmanageable. This cognitive attribute serves to heavily influence the development of exaggerated apprehension, unease, and evasive conduct, which are commonly regarded as hallmark symptoms of anxiety disorders [9]. Given the acknowledgement that adult separation anxiety disorder falls under the anxiety disorder spectrum, it is postulated that maladaptive attitudes should not be disregarded and must be taken into consideration.

There is a possibility that the stressors experienced during pregnancy may contribute to the development of separation anxiety. As far as we know, there hasn't been any research on the topic of adult separation anxiety and dysfunctional attitudes. The purpose of this research was to examine the prevalence of adult separation anxiety in women during pregnancy and its connection to dysfunctional attitudes from the perspective of cognitive theory.

## Materials and methods

Participants

Pregnant women who applied to a state hospital gynaecology and obstetrics clinic between May 2021 and September 2021 were randomly evaluated. The research only included pregnant women over the age of 18 who provided written permission. The research excluded illiterate women with a history of mental illness and its treatment. In addition, those with organic pathology affecting the central nervous system were not included in the study. In the research, 206 pregnant women were included. Sixteen pregnant women were removed from the research because of incomplete questionnaires or scales. In total, 190 pregnant women participated in the research. Ethics committee approval was obtained on April 2, 2021, from the Afyonkarahisar University of Health Sciences Ethics Committee, Afyonkarahisar, Türkiye, for this study (Approval no. 2021/4/237). Patients were informed of the study's purpose and procedures before giving their written permission.

Material

In the research, the sociodemographic forms Adult Separation Anxiety Questionnaire (ASA-27), and Dysfunctional Attitudes Scale Short Form-Revised (DAS-R) were utilised to assess participants.

Sociodemographic form

The participants were questioned on a questionnaire created by the researchers about their age, education, marital status, occupation, number of pregnancies, gestational week, history of miscarriages, and the death of a child.

Adult Separation Anxiety Questionnaire (ASA-27)

It is a self-reported scale of the Likert type that measures separation anxiety symptoms and symptom intensity [10]. This 27-item scale has a total score ranging from 0 to 81. The threshold was set at 25. A high score implies that separation anxiety is becoming more severe. There have been validity and reliability studies conducted in Turkish [11].

Dysfunctional Attitudes Scale-Revised (DAS-R)

In 1978, Weissman and Beck created the Dysfunctional Attitude Scale. The Turkish reliability and validity scores of the Short Form were evaluated by Batmaz and Özdel. It is a self-reported Likert-type scale consisting of 13 items. The scale does not have a cutoff score, and high scores are associated with dysfunctional attitudes. It consists of two subscales: perfectionism/success and the need for approval/dependence. The first eight items are related to the perfectionism/success subscale, while the last five items are related to the need for approval/dependency subscale [9].

Statistical analysis

Analyses were performed using IBM Statistical Package for Social Sciences (SPSS) version 26.0 software (IBM Corp., Armonk, NY). Histograms, Kolmogorov-Smirnov, and Shapiro-Wilk tests were used to determine whether the data were normally distributed. Since the data did not fit the normal distribution, nonparametric tests were performed. The Mann-Whitney U test was used to compare two groups, and values were given as mean ± SD. The Chi-square test was used to determine the relationship between categorical variables, and values were given as n (%). The relationship between variables was analysed by Spearman correlation analysis. The prediction graph of DAS-R and gestational week for predicting adult separation anxiety disorder (ASAD) status was plotted by receiver operating characteristic curve (ROC) analysis. Statistical significance was accepted as two-way and level <0.05.

## Results

The study included 206 pregnant women; 16 pregnant women (7.7%) were excluded from the study due to incomplete questionnaires or scales. The study was conducted with 190 pregnant women. Of all pregnant women, 60 (31.6%) were in the first trimester, 52 (27.4%) in the second trimester, and 78 (41.1%) in the third trimester. According to ASA-27, 45.8% (n=87) of the 190 pregnant participants had significant ASAD symptoms. No significant difference was found between the ASAD (+) and ASAD (-) groups in terms of sociodemographic characteristics such as age, education, marital status, employment status, economic status, number of pregnancies, miscarriage, child death, pregnancy planning, number of children, gestational period, and mode of delivery (p>0.05). However, a significant relationship was found between the gestational period and the presence of ASAD (p=0.02). The presence of ASAD was found to be higher in the third trimester compared to the second trimester (Table 1).

**Table 1:** Evaluating sociodemographic characteristics according to the existence of ASAD Statistical significance is shown in bold. Chi-square test n (%) ASAD: adult separation anxiety disorder; IVF: in vitro fertilization

	ASAD(+) (n=87)	ASAD (-) (n=103)	x²	p
Age			1.773	.860
Below 24 years	43 (49.4%)	57 (55.3%)		
Between 25-29 years	21 (24.1%)	22 (21.4%)		
Between 30-34 years	15 (17.2%)	17 (16.5%)		
Between 35-39 years	7 (8.0%)	7 (6.8%)		
Over 40 years	1 (1.1%)	0 (0.0%)		
Education			2.013	.837
Literate	1 (1.1%)	1 (1.0%)		
Primary education	39 (44.8%)	41 (39.8%)		
High school	32 (36.8%)	42 (40.8%)		
University	14 (16.1%)	19 (18.4%)		
Master (PhD)	1 (1.1%)	0 (0.0%)		
Marital status			1.047	1.000
Married	86 (98.9%)	101 (98.1%)		
Single	1 (1.1%)	1 (1.0%)		
Divorced	0 (0.0%)	1 (1.0%)		
Employment status			.178	.821
Working	11 (12.6%)	11 (10.7%)		
Not working	76 (87.4%)	92 (89.3%)		
Economical situation			5.065	.258
Low income	12 (13.8%)	10 (9.7%)		
Below middle class	17 (19.5%)	12 (11.7%)		
Middle class	56 (64.4%)	74 (71.8%)		
Upper middle class	2 (2.3%)	5 (4.9%)		
Wealthy	0 (0.0%)	2 (1.9%)		
Number of pregnancies			1.774	.624
First	28 (32.2%)	42 (40.8%)		
Second	24 (27.6%)	27 (26.2%)		
Third	22 (25.3%)	20 (19.4%)		
Four and more	13 (14.9%)	14 (13.6%)		
Abortions			.340	.631
Yes	27 (31.0%)	28 (27.2%)		
No	60 (69.0%)	75 (72.8%)		
Child mortality			1.246	.334
Yes	11 (12.6%)	8 (7.8%)		
No	76 (87.4%)	95 (92.2%)		
Type of pregnancy			.750	.702
Planned pregnancy	64 (73.6%)	79 (76.7%)		
Unexpected pregnancy	21 (24.1%)	23 (22.3%)		
IVF	2 (2.3%)	1 (1.0%)		
Number of children			2.320	.728
0	50 (57.5%)	55 (53.4%)		
1	18 (20.7%)	30 (29.1%)		
2	13 (14.9%)	13 (12.6%)		
3	5 (5.7%)	4 (3.9%)		
≥4	1(1.1%)	1(1.0%)		
Gestation period			12.010	0.02
First trimester	19 (21.8%)	41 (39.8%)		
Second trimester	21 (24.1%)	31 (30.1%)		
Third trimester	47 (54.0%)	31 (30.1%)		
Type of labour			.400	.806
No labour	35 (40.2%)	46 (44.7%)		
Cesarean section	23 (26.4%)	26 (25.2%)		
Normal vaginal delivery	29 (33.3%)	31 (30.1%)		

**Figure 1:**
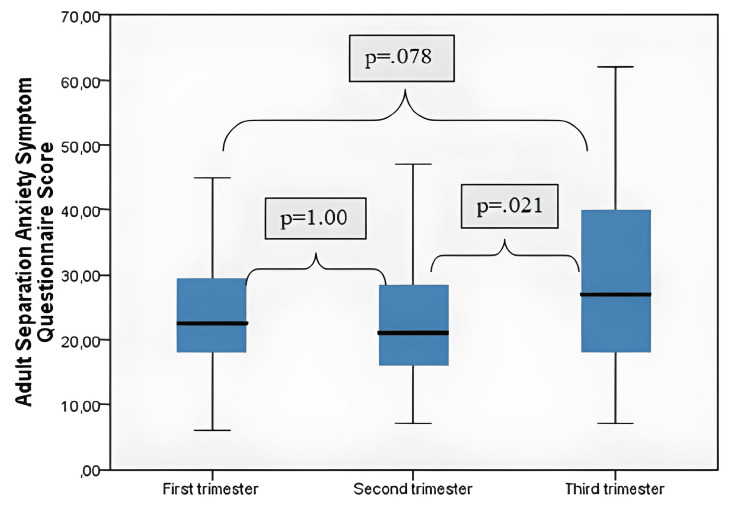
Evaluation of adult separation anxiety questionnaire scores between trimesters

The ASA-27 scores in all three trimesters of pregnancy were compared, and the mean ASA-27 scores of pregnant women in the second trimester were significantly lower than those in the third trimester (p=.021) (Figure 1).

Total DAS-R and subscale scores were compared according to ASAD positivity. The total DAS-R score, need for approval/dependency, and perfectionism/achievement subscale scores were all higher in those with significant ASAD symptoms (u=2466.500 and p<0.001 u=2514.500; and p<0.001 u=2848.500; and p<0.001. respectively) (Table 2).

**Table 2:** Evaluation of DAS-R and subgroup scores according to the presence of ASAD Statistical significance is shown in bold. ASAD: adult separation anxiety disorder; DAS-R: Dysfunctional Attitudes Scale-Revised

	ASAD(+) (n=87) Mean±SD	ASAD(-) (n=103) Mean±SD	U value	p
DAS-R				
Perfectionism/success	19.41 ± 7.64	14.78 ± 7.55	2514.5	.0001
Need for approval/dependency	12.55 ± 6.08	9.26 ± 4.48	2848.5	.0001
Total	31.96 ± 12.49	24.04 ± 10.89	2466.5	.0001

There was a high and positive correlation between the DAS-R and perfectionism/achievement (r = .941, p=.001). There was a high and positive correlation between DAS-R and the need for approval/dependency (r =.874, p= .001). A moderate positive correlation was observed between DAS-R and the ASA-27 total score (r = .415, p=.001). There was no significant correlation between DAS-R and gestational week (r = -.059, p = .417) (Table 3).

**Table 3:** Correlation between DAS-R, perfectionism/achievement, need for approval/ dependency, ASA total score, and gestational week DAS-R: Dysfunctional Attitudes Scale-Revised; ASA: adult separation anxiety ** : Correlation is significant at the 0.01 level. * : Correlation is significant at the 0.05 level.

	1	2	3	4	5
1. DAS-R	1				
2. Perfectionism/success	.941**	1			
	.0001				
3. Need for approval/dependency	.874**	.658**	1		
	.0001	.0001			
4. ASA total score	.415**	.368**	.396**	1	
	.0001	.0001	.0001		
5. Gestational week	-.059	-.088	-.005	.153*	1
	.417	.226	.944	.035	

**Figure 2:**
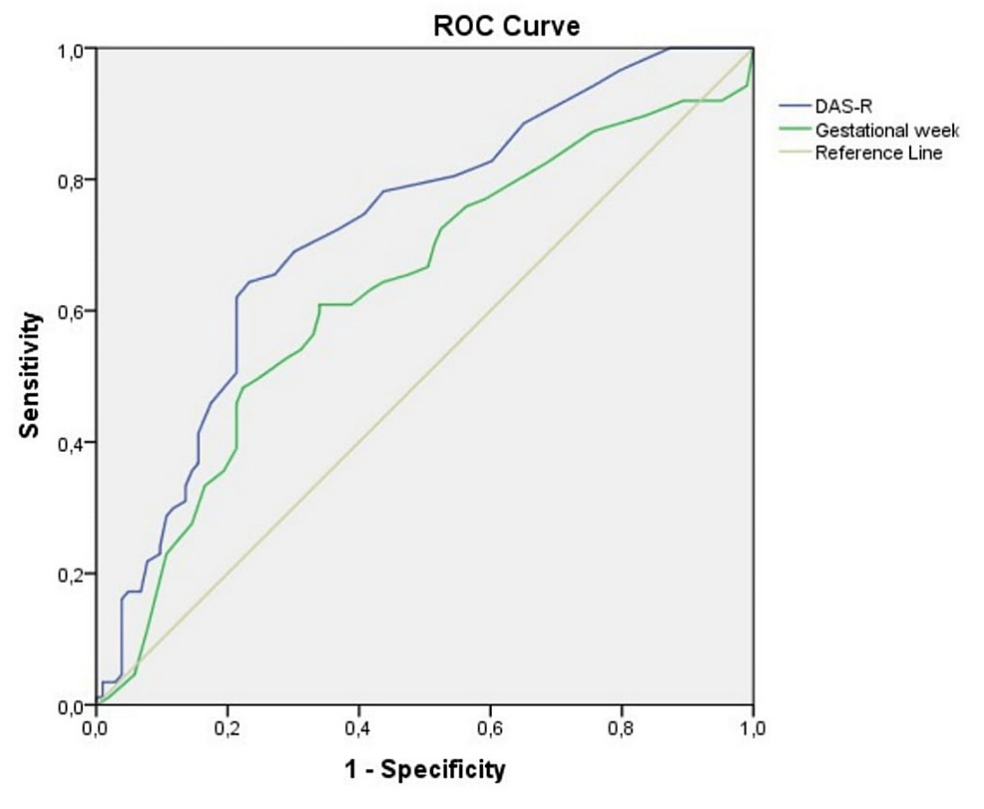
ROC analysis of DAS-R and gestational week for ASAD positivity

A ROC analysis was performed to evaluate the performance of DAS-R and gestational week in predicting ASAD positivity (Figure 2).

When the cut-off point for the DAS-R value was set at 25, the sensitivity and specificity were 68.97% and 69.90%, respectively. Furthermore, the positive and negative predictive values were 65.93% and 72.73%, respectively. The AUC value was calculated at 0.725. Similarly, when a cut-off point of 24 was set for gestational week, the sensitivity and specificity were 60.92% and 66.02%, respectively. Positive and negative predictive values were 59.77% and 66.67%, respectively, with an AUC of 0.632 (Table 4).

**Table 4:** Predicted values of DAS-R and gestational week for ASAD positivity as a result of ROC analysis PPV: positive predictive value; NPV: negative predictive value; AUC: area under the ROC curve; ROC: receiver operating characteristic curve; DAS-R: Dysfunctional Attitudes Scale-Revised; ASAD: Adult separation anxiety disorder

Cutpoint	Sensitivity (%)	Specificity (%)	PPV (%)	NPV (%)	AUC
DAS-R					
25	68.97%	69.90%	65.93%	72.73%	0.725
Gestational week					
24	60.92%	66.02%	59.77%	66.67%	0.632

## Discussion

We found that 45.8% of pregnant women suffer from separation anxiety at some point throughout their pregnancy. Significant ASAD symptoms were associated with elevated scores on the DAS-R total, perfectionism, and addiction anxiety subscales. This rate is about the same as what the research says about the rate of separation anxiety in pregnant women. The reasons for its higher incidence than in the general population may be differences in education levels, cultural differences, psychiatric comorbidities, and the presence of the female gender [12,13].

In the literature, it has been said that 6.6% of adults have separation anxiety disorder at some point in their lives [6]. Pregnancy predisposes to the development of mental illnesses. The most frequent mental disorders during pregnancy are anxiety and depressive disorders [2,3]. The number of studies investigating the frequency of separation anxiety during pregnancy is limited. In a study conducted with 127 pregnant women in 2014, the rate of women showing signs of adult separation anxiety disorder during pregnancy was approximately 45% [13]. In the study by Degirmenci et al. conducted with 297 pregnant women in 2020, the rate of adult separation anxiety disorder was found to be 56.2% [12].

It is recognised that early pregnancy is a risk factor for the development of anxiety symptoms in women. In our research, however, there was no correlation between the age of the patients and the prevalence of separation anxiety disorder. In a study investigating the frequency of adult separation anxiety during pregnancy, no relationship was found between age and adult separation anxiety [14].

In our research, no connection between the prevalence of separation anxiety disorder and the number of pregnancies was shown to be statistically significant. There are studies in the literature that, like ours, show no connection between the number of pregnancies [15,16].

When we compared separation anxiety levels between trimesters in our study, we discovered that the third trimester separation anxiety score was higher than the second trimester. Anxiety in pregnant women was studied in 2009, and the results showed that it was highest in the first and third trimesters and lowest in the second [17]. Değirmenci et al. Similar to our research, a substantial difference was reported between the second and third trimesters for separation anxiety [12].

In cognitive theory, dysfunctional attitudes are defined as negative beliefs that a person develops against himself, his social environment, and the outside world during communication with others. In the research, it has been shown that there is a relationship between dysfunctional attitudes and depression and anxiety [9,18]. It is known that there is a close relationship between emotional responses to events and cognitive processes in anxiety disorder [19]. There are many studies examining the relationship between anxiety disorders, especially depression, and dysfunctional attitudes. With the recognition that adult separation anxiety disorder is part of the spectrum of anxiety disorders, it is thought that dysfunctional attitudes cannot be ignored. So far as we know, our study is the first to look into the link between separation anxiety and dysfunctional attitudes in pregnant women.

The group with a high ASAD score had significantly higher total DAS-R, perfectionism, and addiction anxiety subscale scores. In a study conducted in 2020, the relationship between dysfunctional attitudes and generalised anxiety tendencies was examined. In this study, in which 474 people participated, it was stated that there was a positive and significant relationship between anxiety and dysfunctional attitudes and that dysfunctional attitudes predicted generalised anxiety statistically [20]. In 2019, Parim et al. examined the relationship between anxiety symptoms, automatic thoughts, and dysfunctional attitudes in university students. As a result of the study, it was stated that there is a positive relationship between anxiety and both automatic thoughts and dysfunctional attitudes [21]. Similarly, in a study conducted with 281 people in 2018, the positive association between anxiety levels and dysfunctional attitudes was emphasised [22]. In a study conducted with patients with irritable bowel syndrome in 2010, anxiety disorders were found at a rate of approximately 30%. It has been reported that patients with high anxiety levels also have more dysfunctional attitudes [23]. In a study conducted in 2008, it was reported that test anxiety and dysfunctional attitudes are related [24].

The limitations of our study are that there was no structured interview while evaluating the participants, that it was a cross-sectional study, and that the presence of comorbid conditions was not evaluated. This situation causes a limitation in explaining the relationship between the variables in our study.

## Conclusions

Dysfunctional attitudes are addressed in cognitive behavioural therapy for depression and anxiety disorders. The detection of dysfunctional attitudes in adult separation anxiety may guide treatment in cognitive behavioural therapy. We believe that the association between adult separation anxiety and dysfunctional attitudes should be proven in larger-sample clinical research.
